# Comparison of Structural and Functional Properties of Wheat Starch Under Different Soil Drought Conditions

**DOI:** 10.1038/s41598-017-10802-3

**Published:** 2017-09-26

**Authors:** Weiyang Zhang, Junfei Gu, Zhiqin Wang, Cunxu Wei, Jianchang Yang, Jianhua Zhang

**Affiliations:** 1 Jiangsu Key Laboratory of Crop Genetics and Physiology/Co-Innovation Center for Modern Production Technology of Grain Crops, Yangzhou University, Yangzhou, Jiangsu China; 2 School of Life Sciences and State Key Laboratory of Agrobiotechnology, The Chinese University of Hong Kong, Hong Kong, China

## Abstract

Drought influences cereal crop yield and quality. However, little is known about changes in the structural and functional properties of wheat starch under soil drought conditions. In this study, two wheat cultivars were subjected to well-watered (WW), moderate soil-drought (MD), and severe soil-drought (SD) from 7 tillers in the main stem to maturity. The structural and functional properties of the resultant endosperm starch were investigated. In comparison with WW soil, the MD increased starch accumulation in grains, the proportion of large starch granules, amylose and amylopectin long branch chain contents, and average amylopectin branch chain length, which were accompanied by the increase in activities of granule bound starch synthase and soluble starch synthase. MD treated-starch had a lower gelatinization enthalpy, and swelling power, but a higher gelatinization temperature, retrogradation enthalpy, and retrogradation percentage when compared to WW conditions. The MD also increased starch resistance to acid hydrolysis, amylase hydrolysis, and *in vitro* digestion. The SD had the opposite effects to the MD in all cases. The results suggest that soil drought more severely affects amylose synthesis than amylopectin synthesis in wheat grains, and moderate soil-drought improves molecular structure and functional properties of the starch.

## Introduction

Wheat (*Triticum aestivum* L.) is an important cereal crop and a staple food for humans and animals worldwide. Starch is the major storage compound in wheat endosperm, accounting for 65–75% of the final dry weight of a grain, and is synthesized in the amyloplast of endosperm cells since 4 days after anthesis (DAA) and the endosperm structure no longer changes after 33 DAA^1,2^. Starch is mainly composed of linear amylose and highly branched amylopectin, which assemble to form a semicrystalline granule^3^. For linear amylose, the glucose units are joined through α-(1,4)-glycosidic linkages which are mainly catalyzed and elongated by granule-bound starch synthase (GBSS). Amylopectin mainly consists of long chains of α-(1,4)-linked D-glucopyranosyl units with occasional branching α-(1,6)-linkages that form branched structure. The α-(1,6)-glycosidic linkages are catalyzed and elongated by starch branching enzymes (SBE) and soluble starch synthase (SSS), respectively^1,4^. Amylose content and amylopectin fine structure greatly influence physicochemical properties that affect grain quality, flour quality, and starch properties^4^. Grain quality is a complex trait with various determinants, including physical appearance, nutritional value, and eating and cooking quality. These factors are important for consumers, and are associated with the physicochemical properties of crop starch, including hydration, gelatinization, volume expansion, and digestion properties^5,6^. Furthermore, apparent amylose content, pasting viscosity characteristics, gel texture, thermal and retrogradation properties, and amylose and amylopectin fine structures have been established to precisely evaluate the quality of grain and starch-based foods^7,8^.

It is well known that seed yield and quality are determined both genetically and environmentally^9^. Soil water status, especially during the grain development, probably ranks as the most important environmental factor affecting grain yield and quality in cereals^10,11^. The arid and semiarid rangelands exist all over the world, such as in the Middle East and North Africa, South and Central Asia, South and North America^10^. Various physiological and chemical reactions can be activated when plants are subjected to water stress during various developmental stages^12,13^. Water stress can affect starch synthesis and grain weight and can change the components and accumulation rate of grain starch^14,15^. Crop response to water stress varies with water conservation strategies, for instance, moderate soil drought could accelerate plant growth or development, whereas severe soil drought could cause programmed cell death^16,17^.

Previous studies have mainly focused on the effects of drought on grain development^18–22^, starch biosynthesis^12,23^ and physicochemical properties^11,24,25^ in cereal crops. However, limited information is available regarding the influence of soil drought on the functional or fine structural characteristics of endosperm starch and the underlying physiological mechanism. Therefore, in this study, fine structures and functional properties of wheat starch were determined and carefully compared in two wheat cultivars under different degrees of soil drought. The objectives of this study were (1) to investigate how soil drought affects physicochemical properties of starches in wheat kernels, (2) to elucidate the relationship between functional properties and fine structures of starch, and (3) to test the hypothesis that moderate soil drought increases grain quality by improving structural and functional properties of starch in the kernel.

## Results

### Leaf water potential, grain filling and grain yield

**Figure 1:**
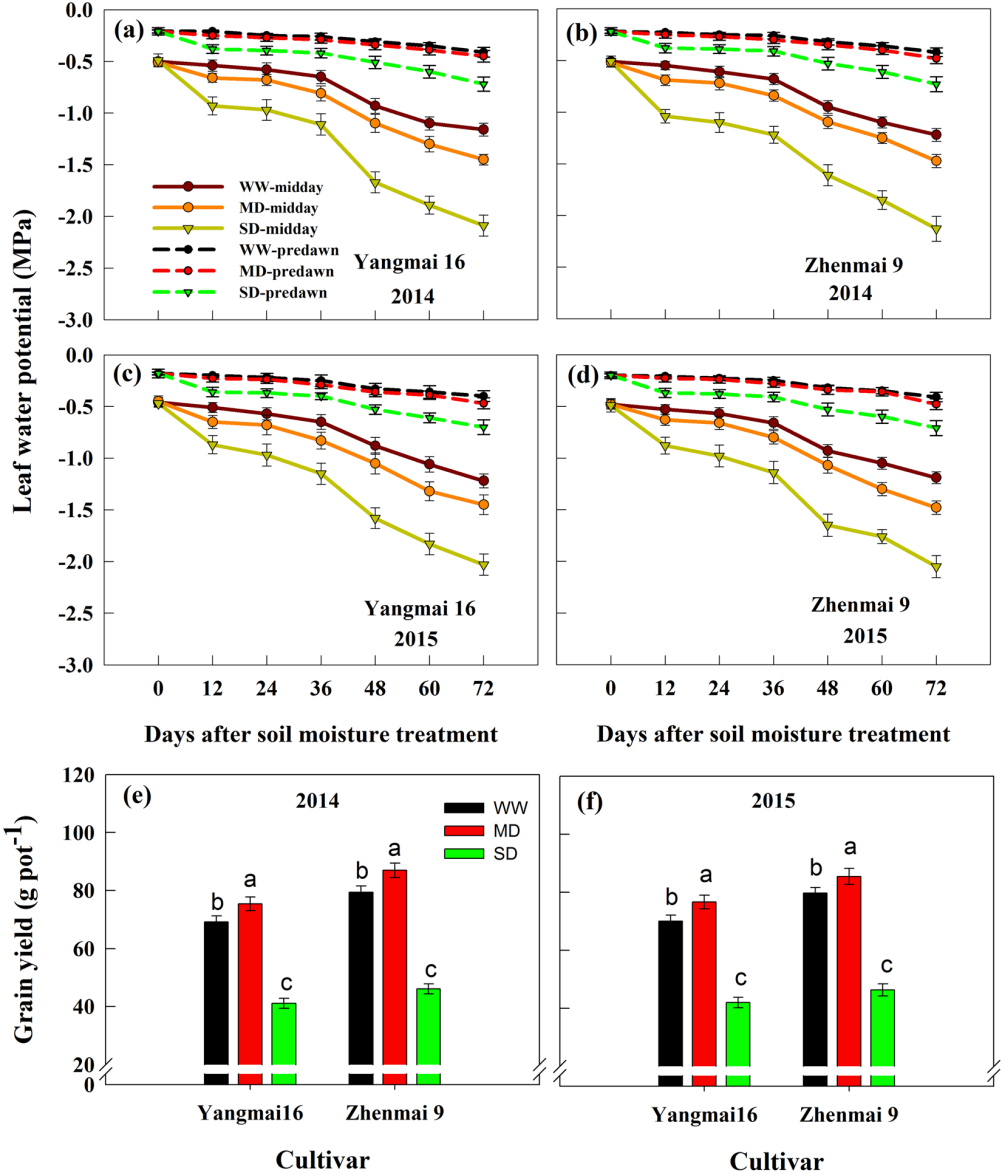
Leaf water potential (**a**–**d**) and grain yield (**e**,**f**) of the wheat cultivars Yangmai 16 and Zhenmai 9 under various soil moisture treatments in 2014 and 2015. WW, MD, and SD represent well-watered, moderate soil-drought, and severe soil-drought treatments, respectively. Vertical bars represent ± standard deviations of the mean (n = 6 for Leaf water potential and n = 10 for grain yield), where they exceed the size of the symbol.

The leaf water potential (LWP) gradually decreased during the growing season (Fig. 1a–d). For plants grown under the well-watered (WW) treatment, midday (11:30) LWP ranged from −0.46 MPa at the beginning of measurements to −1.19 MPa at the late grain filling stage. The LWP (11:30) was reduced under soil drought treatments, and ranged from −0.45 MPa to −1.48 MPa under moderate soil-drought (MD) and from −0.49 MPa to −2.03 MPa under severe soil-drought (SD). In addition, the predawn (06:00 h) LWP for MD plants was not significantly different from that for WW plants, but was significantly lower for SD plants, indicating MD plants could rehydrate overnight, whereas SD plants could not. The results indicate that moderate soil-drought during the grain-filling period would not seriously affect the plant water status. Both cultivars exhibited similar changes.

**Figure 2:**
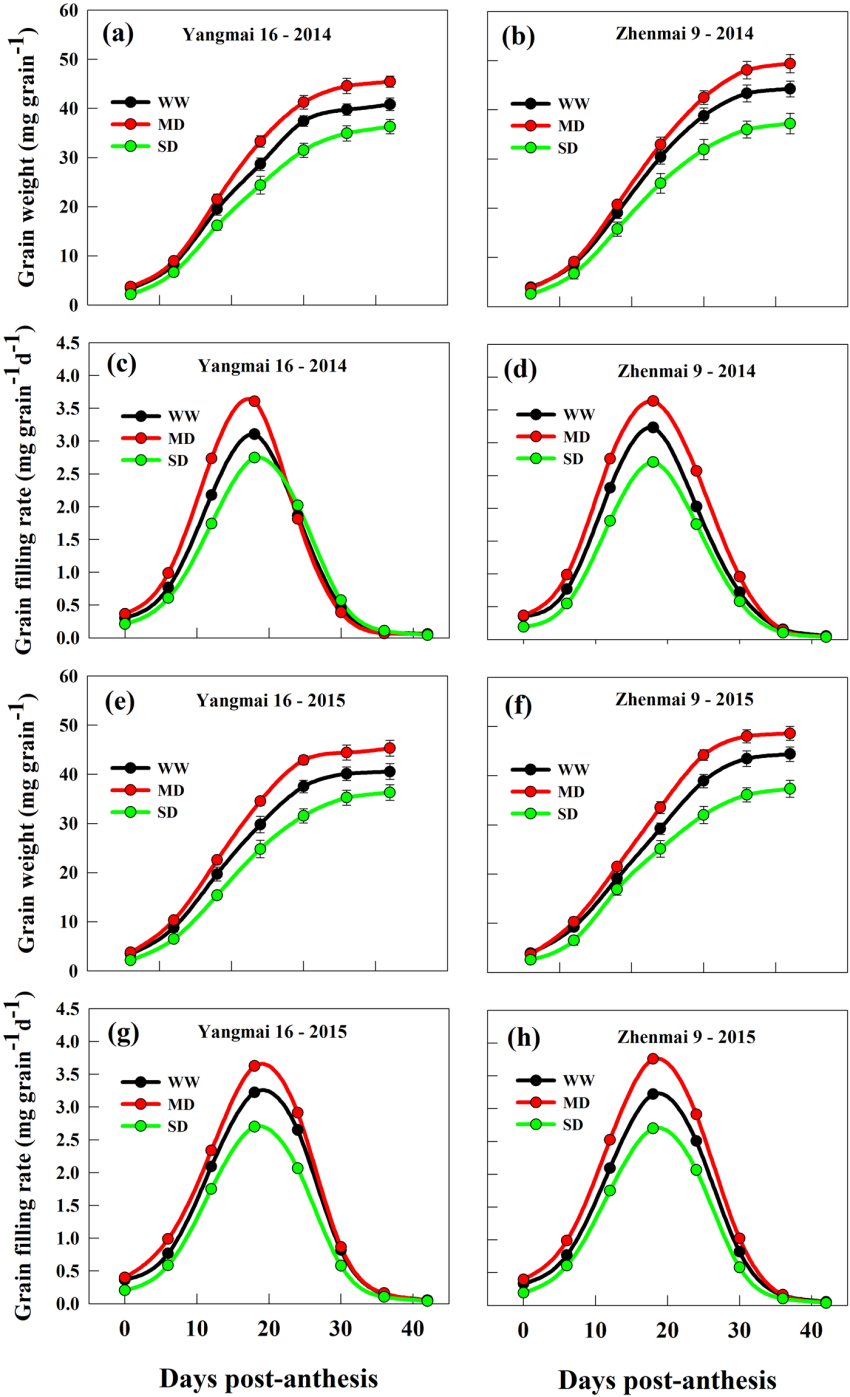
Grain weight (**a**,**b**,**e**,**f**) and grain filling rate (**c**,**d**,**g**,**h**) in grains of wheat cultivars Yangmai 16 and Zhenmai 9 under various soil moisture treatments during grain filling in 2014 and 2015. WW, MD, and SD represent well-watered, moderate soil-drought, and severe soil-drought treatments, respectively. Vertical bars represent ± standard deviations of the mean (n = 3), where they exceed the size of the symbol.

Soil drought greatly affected grain weight and grain yield of both cultivars (Figs 1e,f and 2a–h). The grain filling rate and grain weight were significantly increased under the MD compared with those under the WW; however, the SD markedly decreased grain filling rate and grain weight of the both cultivars (Fig. 2a–h). As a result, the MD significantly increased, whereas the SD decreased, grain yield relative to the WW treatment (Fig. 1e,f).

### Activities of enzymes

**Figure 3:**
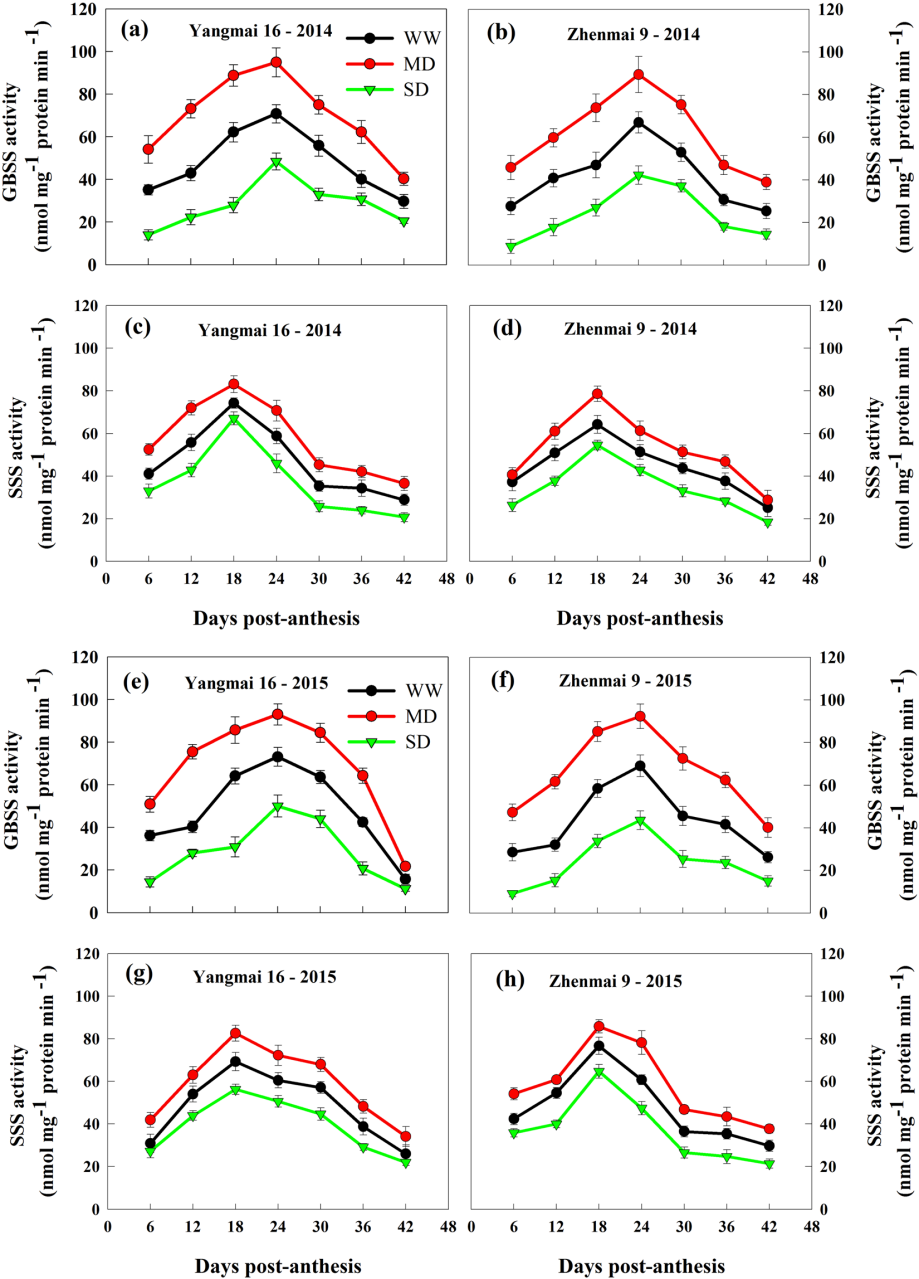
Changes in activities of GBSS (**a**,**b**,**e**,**f**) and SSS (**c**,**d**,**g**,**h**) in grains of wheat cultivars Yangmai 16 and Zhenmai 9 under various soil moisture treatments during grain filling in 2014 and 2015. WW, MD, and SD represent well-watered, moderate soil-drought, and severe soil-drought treatments, respectively. Vertical bars represent ± standard deviations of the mean (n = 3), where they exceed the size of the symbol.

Changes in activities of soluble starch synthase (SSS) and granule bound starch synthase (GBSS) in grains under various soil moisture treatments during grain filling were shown in Fig. 3. Both GBSS and SSS activities were significantly increased by the MD, whereas were decreased by the SD. Substantial change in GBSS activity was observed, in contrast a slight change in SSS activity under soil drought conditions (Fig. 3a–f).

### Total starch accumulation and starch granule size distribution

**Figure 4:**
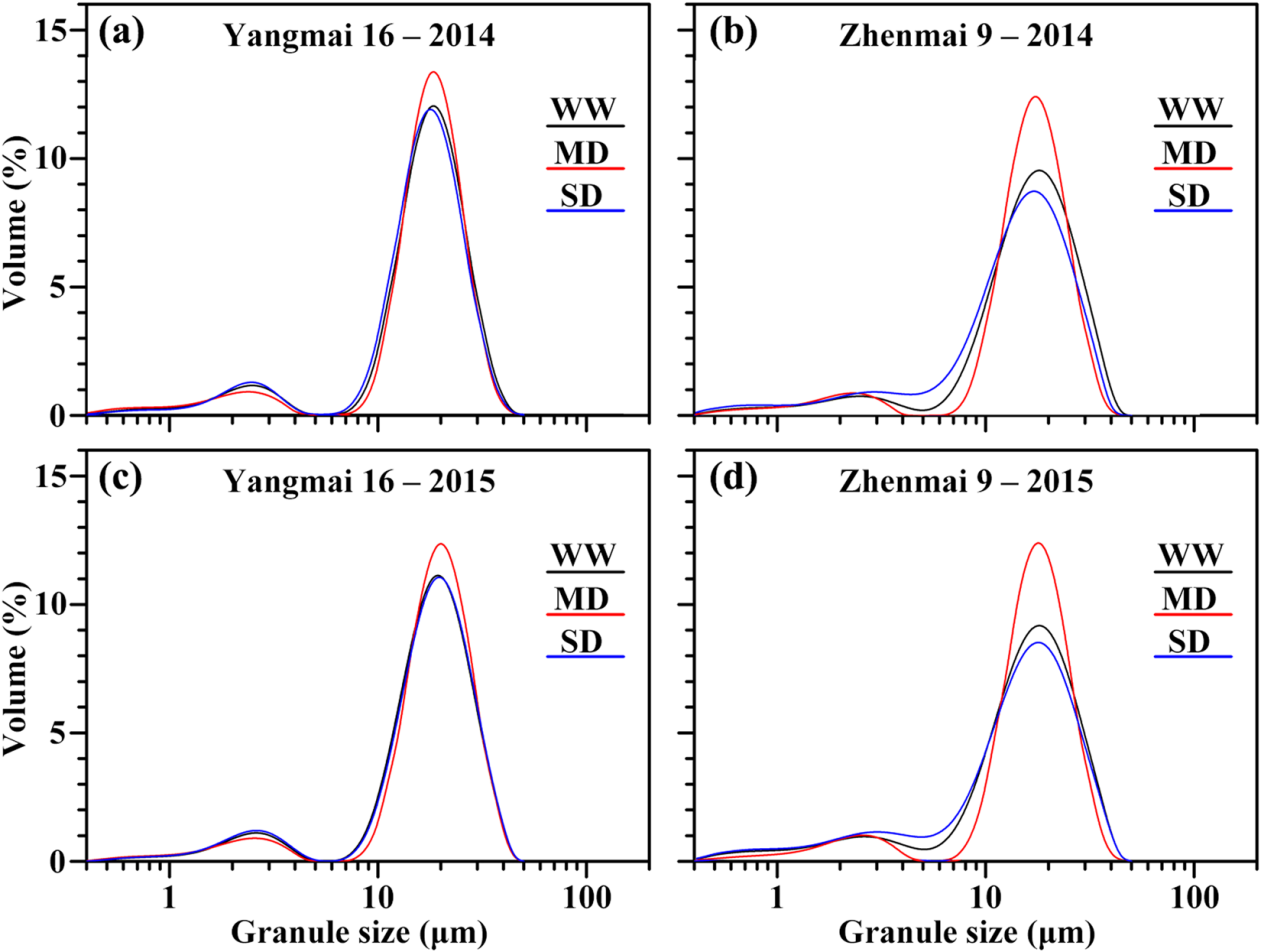
Granule size distribution in starch of wheat cultivars Yangmai 16 and Zhenmai 9 under various soil moisture treatments in 2014 (**a**,**b**) and 2015 (**c**,**d**). WW, MD, and SD represent well-watered, moderate soil-drought, and severe soil-drought treatments, respectively.

Compared with that in the WW grains, the total starch accumulation was significantly higher in MD-treated grains and markedly lower in SD-treated grains (Table 1). The proportion of large and small granules varied with soil moisture treatments (Fig. 4 and Table 1). Compared with the WW treatment, the MD treatment had significantly more large granules and fewer small granules, while the opposite occurred under the SD treatment (Table 1). The proportion of large granules in the wheat grains was in the range of 70.55–89.15% (Table 1), suggesting that the large granules contribute the majority of the volume of starch granules in wheat endosperm.

**Table 1:** Total starch accumulation in grains and granule size distribution in wheat starches under various soil moisture treatments^a^.

Year/Cultivar	Treatment	Starch accumulation (mg grain^−1^)	Volume (%)	
			Large granules (diameter > 10.0 μm)	Small granules (diameter < 10.0 μm)
2014				
Yangmai16	WW	33.14 ± 0.41b	86.14 ± 1.13b	13.86 ± 0.13b
	MD	36.92 ± 0.15a	88.58 ± 0.90a	11.42 ± 0.90c
	SD	29.98 ± 1.01c	84.81 ± 1.09c	15.19 ± 1.09a
Zhenmai 9	WW	31.17 ± 0.42b	79.58 ± 1.06b	20.42 ± 1.06b
	MD	34.76 ± 0.70a	85.37 ± 0.99a	14.63 ± 0.99c
	SD	27.54 ± 0.75c	71.17 ± 1.08c	28.83 ± 1.08a
2015				
Yangmai 16	WW	33.83 ± 0.37b	86.07 ± 0.70b	13.93 ± 0.70a
	MD	37.29 ± 0.86a	89.15 ± 1.04a	10.85 ± 1.04b
	SD	30.65 ± 0.35c	85.55 ± 1.40b	14.45 ± 1.40a
Zhenmai 9	WW	30.39 ± 1.00b	76.55 ± 1.00b	23.45 ± 1.00b
	MD	33.45 ± 1.22a	86.19 ± 0.83a	13.81 ± 0.83c
	SD	26.95 ± 0.31c	70.55 ± 1.23c	29.45 ± 1.23a
Analysis of variance				
Year (Y)		0.46^ns^	0.57^ns^	0.57^ns^
Cultivar (C)		151.86^**^	587.19^**^	587.19^**^
Treatment (T)		274.85^**^	236.70^**^	236.70^**^
Y × C		9.64^**^	3.76^ns^	3.76^ns^
Y × T		0.46^ns^	3.64^*^	3.64^*^
C × T		0.23^ns^	86.26^**^	86.26^**^

^a^Data are means ± standard deviation of three independent measurements, with different letters indicating significant statistical differencesat the p ≤ 0.05 level in the same column and the same cultivar in the same year. *^,^**F values significant at the *P* = 0.05 and *P* = 0.01 levels, respectively. ns denotes insignificant at the *P* = 0.05 level.

### Apparent amylose content and molecular weight distributions of starch

**Figure 5:**
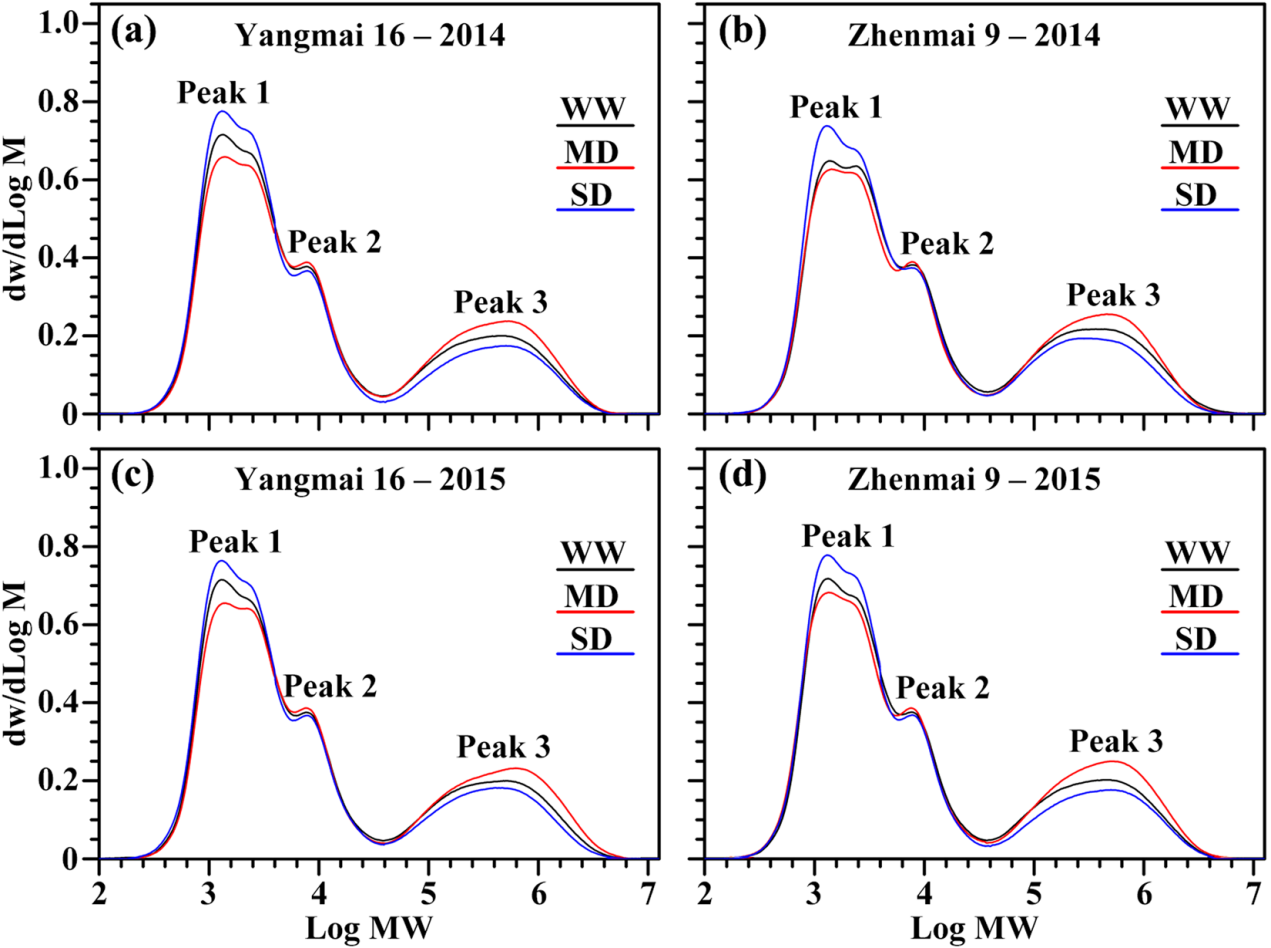
Gel-permeation chromatography (GPC) of isoamylase-debranched starches for wheat cultivars Yangmai 16 and Zhenmai 9 under various soil moisture treatments in 2014 (**a**,**b**) and 2015 (**c**,**d**). WW, MD, and SD represent well-watered, moderate soil-drought, and severe soil-drought treatments, respectively.

The apparent amylose content and molecular weight distributions of wheat starches showed variable responses to the soil moisture treatments (Table 2 and Fig. 5). The MD treatment significantly increased, while the SD markedly decreased, the apparent amylose content of starches for both cultivars (Table 2).

**Table 2:** Apparent amylose contents and molecular weight distributions in starches under various soil moisture treatments^a^.

Year/Cultivar	Treatment	Apparent amylose content (%)^b^	Peak area from gel-permeation chromatography			
			Peak 1 (%)	Peak 2 (%)	Peak 3 (%)	Peak 1/Peak 2
2014						
Yangmai 16	WW	28.31 ± 0.51b	57.62 ± 0.58b	16.34 ± 0.34ab	26.05 ± 0.24b	3.53 ± 0.11b
	MD	33.15 ± 1.46a	53.79 ± 0.55c	16.91 ± 0.10a	29.30 ± 0.45a	3.18 ± 0.05c
	SD	24.61 ± 0.81c	61.73 ± 0.27a	15.75 ± 0.42b	22.52 ± 0.21c	3.92 ± 0.12a
Zhenmai 9	WW	32.88 ± 0.90b	54.76 ± 0.17b	16.66 ± 0.38b	28.58 ± 0.29b	3.29 ± 0.08b
	MD	35.44 ± 1.26a	50.79 ± 0.37c	17.74 ± 0.37a	31.47 ± 0.72a	2.86 ± 0.04c
	SD	26.42 ± 0.58c	60.98 ± 0.67a	14.73 ± 0.46c	24.28 ± 0.42c	4.14 ± 0.16a
2015						
Yangmai 16	WW	29.25 ± 1.13b	57.66 ± 0.14b	16.05 ± 0.35ab	26.29 ± 0.21b	3.59 ± 0.09ab
	MD	33.53 ± 1.20a	53.41 ± 0.41c	16.54 ± 0.07a	30.04 ± 0.43a	3.23 ± 0.02b
	SD	24.76 ± 1.40c	61.60 ± 0.87a	15.66 ± 0.62b	22.74 ± 0.41c	3.94 ± 0.19a
Zhenmai 9	WW	28.74 ± 0.75b	58.22 ± 0.51b	16.32 ± 0.31b	25.46 ± 0.41b	3.57 ± 0.09b
	MD	34.81 ± 1.47a	52.60 ± 0.36c	17.29 ± 0.70a	30.11 ± 0.49a	3.05 ± 0.14c
	SD	24.35 ± 1.14c	62.25 ± 0.42a	15.92 ± 0.15b	21.83 ± 0.42c	3.91 ± 0.05a
Analysis of variance						
Year (Y)		182.97^**^	39.28^**^	0.18^ns^	47.66^**^	2.65^ns^
Cultivar (C)		425.52^**^	40.92^**^	3.11^ns^	33.37^**^	6.67^*^
Treatment (T)		73.50^**^	1032.05^**^	48.71^**^	948.79^**^	198.15^**^
Y × C		410.94^**^	51.90^**^	2.08^ns^	95.81^**^	0.19^ns^
Y × T		300.30^**^	5.28^*^	5.31^*^	5.88^**^	5.40^*^
C × T		276.75^**^	11.15^**^	6.45^**^	2.15^ns^	7.58^**^

^a^Data are means ± standard deviation of three independent measurements, with different letters indicating significant statistical differences at the p ≤ 0.05 level in the same column and the same cultivar in the same year.

^b^Apparent amylose content was determined by iodine adsorption method. *^,^**F values significant at the *P* = 0.05 and *P* = 0.01 levels, respectively. ns denotes insignificant at the *P* = 0.05 level.

In the gel permeation chromatography (GPC) profile of debranched starch, peaks 1 and 2 were designated as different amylopectin branch chains, consisting of short (A and short B chains) and long (long B chains) branch chains, respectively, while peak 3 corresponded with amylose^26^. The weight distributions of the short amylopectin branch chain, long amylopectin branch chain, and amylose were calculated from the areas under peaks 1, 2, and 3, respectively. The extent of amylopectin branching is described by the area ratio of peaks 1 and 2, where a higher ratio indicates a higher degree of branching^27^. The peak areas of wheat starches in GPC are summarized in Table 2. Compared with the WW treatment, the MD treatment significantly increased the amylose content and amylopectin long (long B chains) branch chains, but decreased the short branch chains of amylopectin, for both cultivars. The SD treatment had the opposite effects. The degree of amylopectin branching also differed significantly between starches from soil moisture treatments. MD-treated starch had a significantly lower branching degree, while SD-treated starch had a higher branching degree, than WW starch (Table 2). The apparent amylose content, determined using the iodometric method, was higher than the amylose content determined by GPC (Table 2), due to intermediate and long amylopectin branch chains binding to iodine^28^.

### Chain length distribution of amylopectin

**Figure 6:**
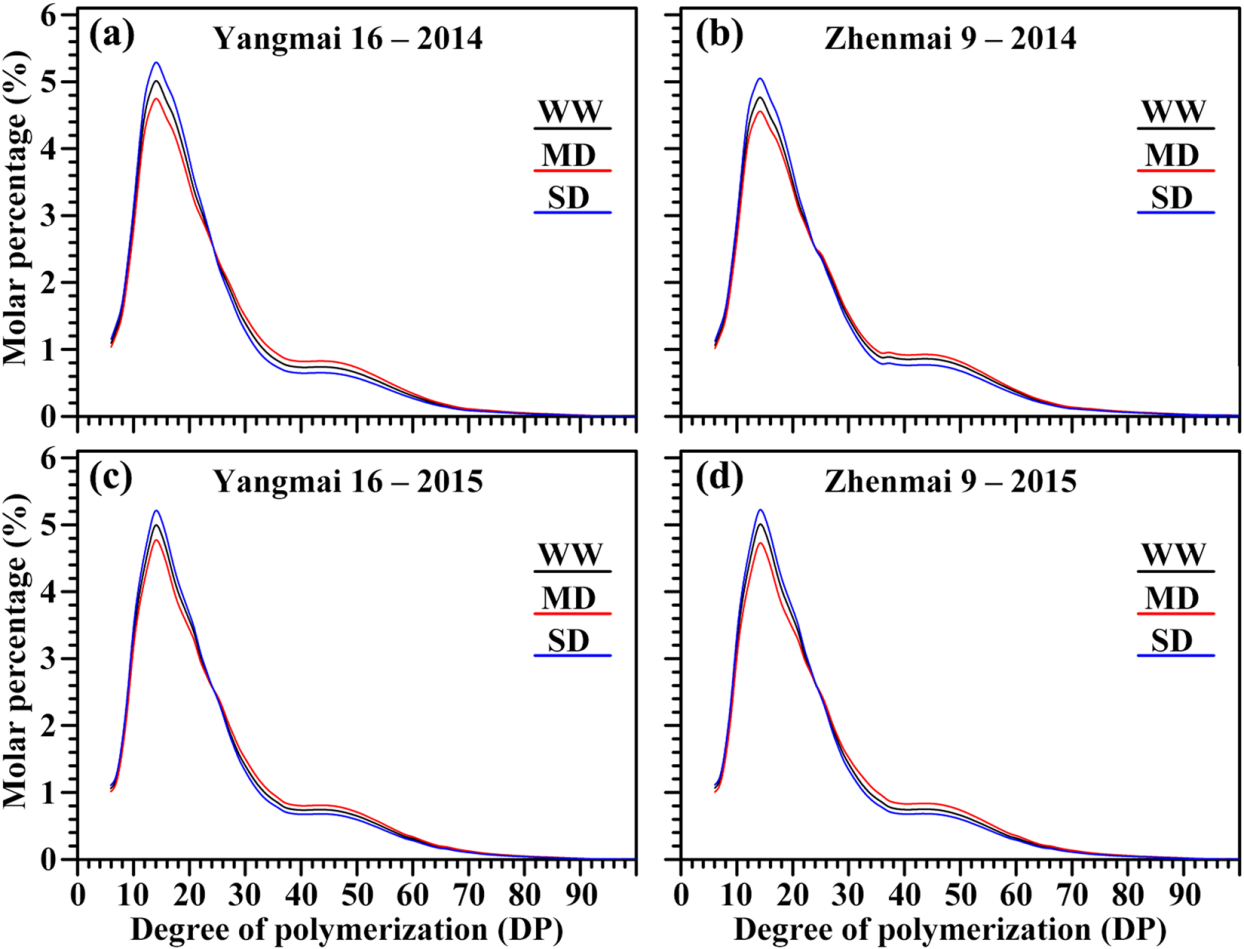
High-performance anion-exchange chromatography (HPAEC) of isoamylase-debranched starches for wheat cultivars Yangmai 16 and Zhenmai 9 under various soil moisture treatments in 2014 (**a**,**b**) and 2015 (**c**,**d**). WW, MD, and SD represent well-watered, moderate soil-drought, and severe soil-drought treatments, respectively.

The amylopectin chain length distribution using high-performance anion-exchange chromatography (HPAEC) is shown in Fig. 6 and Table 3. Amylopectin branch chains are usually classified as A (DP 6-12), B1 (DP 13-24), B2 (DP 25-36), and B3 + chains (DP ≥ 37) according to the degree of polymerization^29^. The chromatograms of starches from different soil moisture treatments showed distinct differences. The percentages of A, B1, B2, and B3 + chains and the amylopectin average chain length in the starches are shown in Table 3. Amylopectin in MD-treated starch contained a lower percentage of short branch chains, a higher percentage of long branch chains, and a higher average chain length relative to those in WW starch, while the SD treatment had the opposite effects, in agreement with the GPC results. Changes in average chain length of starch altered the branching degree of amylopectin under different soil moisture treatments (refer to Table 2).

**Table 3:** Chain length distributions of amylopectin and relative crystallinity of starch under various soil moisture treatments^a^.

Year/Cultivar	Treatment	Chain length distribution (%) ^b^				Average chain length (DP)	Relative crystallinity (%)
		DP 6–12	DP 13–24	DP 25–36	DP ≥ 37		
2014							
Yangmai16	WW	17.47 ± 0.25b	47.85 ± 0.5b	17.13 ± 0.29b	17.55 ± 0.64b	24.37 ± 0.30ab	24.19 ± 0.33b
	MD	16.55 ± 0.40c	45.62 ± 0.36c	18.20 ± 0.39a	19.63 ± 0.22a	24.94 ± 0.24a	23.03 ± 0.38c
	SD	18.44 ± 0.26a	50.18 ± 0.42a	15.91 ± 0.23c	15.47 ± 0.36c	23.43 ± 0.69b	25.91 ± 0.27a
Zhenmai 9	WW	16.77 ± 0.31b	45.39 ± 0.40b	17.71 ± 0.29b	20.13 ± 0.63b	24.75 ± 0.21b	23.61 ± 0.40b
	MD	16.03 ± 0.48c	43.91 ± 0.40c	18.39 ± 0.14a	21.66 ± 0.20a	25.47 ± 0.53a	22.87 ± 0.24c
	SD	17.76 ± 0.13a	47.49 ± 0.36a	16.77 ± 0.31c	17.98 ± 0.37c	23.89 ± 0.34c	25.08 ± 0.25a
2015							
Yangmai 16	WW	17.70 ± 0.26ab	46.91 ± 0.24b	17.57 ± 0.40b	17.82 ± 0.67b	24.41 ± 0.28a	23.77 ± 0.48b
	MD	16.91 ± 0.41b	45.12 ± 0.20c	18.56 ± 0.27a	19.40 ± 0.30a	25.02 ± 0.41a	22.87 ± 0.33c
	SD	18.48 ± 0.46a	48.46 ± 0.13a	16.76 ± 0.27c	16.30 ± 0.62c	23.48 ± 0.17b	25.19 ± 0.32a
Zhenmai 9	WW	17.77 ± 0.32a	46.94 ± 0.12b	17.51 ± 0.53b	17.78 ± 0.36b	24.12 ± 0.30ab	24.10 ± 0.36b
	MD	16.71 ± 0.27b	44.80 ± 0.27c	18.59 ± 0.40a	19.89 ± 0.43a	25.08 ± 0.47a	22.63 ± 0.48c
	SD	18.47 ± 0.17a	48.60 ± 0.26a	16.70 ± 0.19b	16.23 ± 0.44c	23.25 ± 0.49b	25.93 ± 0.25a
Analysis of variance							
Year (Y)		17.60^**^	0.36^ns^	5.71^*^	24.47^**^	3.53^ns^	0.08^ns^
Cultivar (C)		8.03^**^	116.49^**^	5.43^*^	55.42^**^	1.32^ns^	1.12^ns^
Treatment (T)		69.89^**^	415.10^**^	99.13^**^	157.73^**^	49.95^**^	178.73^**^
Y × C		5.98^*^	106.73^**^	6.77^*^	44.77^**^	5.32^*^	11.81^**^
Y × T		0.34^ns^	3.01^ns^	0.51^ns^	1.23^ns^	0.13^ns^	0.52^ns^
C × T		0.01^ns^	0.53^ns^	0.58^ns^	0.01^ns^	0.32^ns^	0.15^ns^

^a^Data are means ± standard deviation of three independent measurements, with different letters indicating significant statistical differences at the p ≤ 0.05 level in the same column and the same cultivar in the same year.

^b^Chain length distribution was determined using high-performance anion-exchange chromatography (HPAEC). *^,^**F values significant at the *P* = 0.05 and *P* = 0.01 levels, respectively. ns denotes insignificant at the *P* = 0.05 level.

### Starch X-ray diffraction pattern

**Figure 7:**
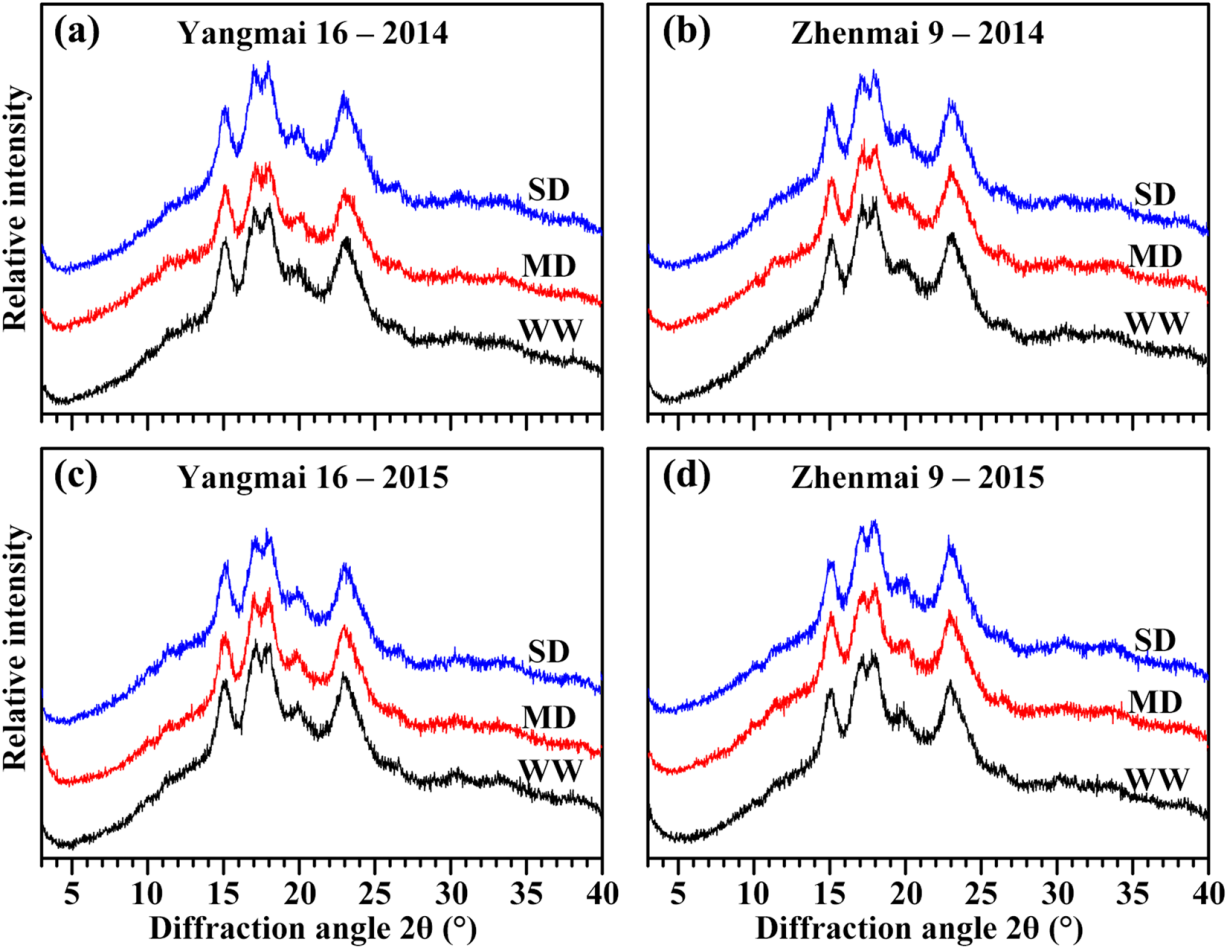
X-ray diffraction patterns for starches of wheat cultivars Yangmai 16 and Zhenmai 9 under various soil moisture treatments in 2014 (**a**,**b**) and 2015 (**c**,**d**). WW, MD, and SD represent well-watered, moderate soil-drought, and severe soil-drought treatments, respectively.

The starch X-ray powder diffraction (XRD) patterns were similar among various soil moisture treatments, and exhibited typical A-type spectra with major diffraction peaks at approximately 15, 17, 18, and 23° (2θ), in agreement with the XRD patterns of normal cereal starches (Fig. 7). The relative crystallinity, calculated from the XRD patterns, showed significant differences among the different soil moisture treatments. The degree of crystallinity in both cultivars decreased under the MD, but increased under the SD, when compared with that under the WW treatment (Fig. 7 and Table 3).

### Starch swelling power

**Figure 8:**
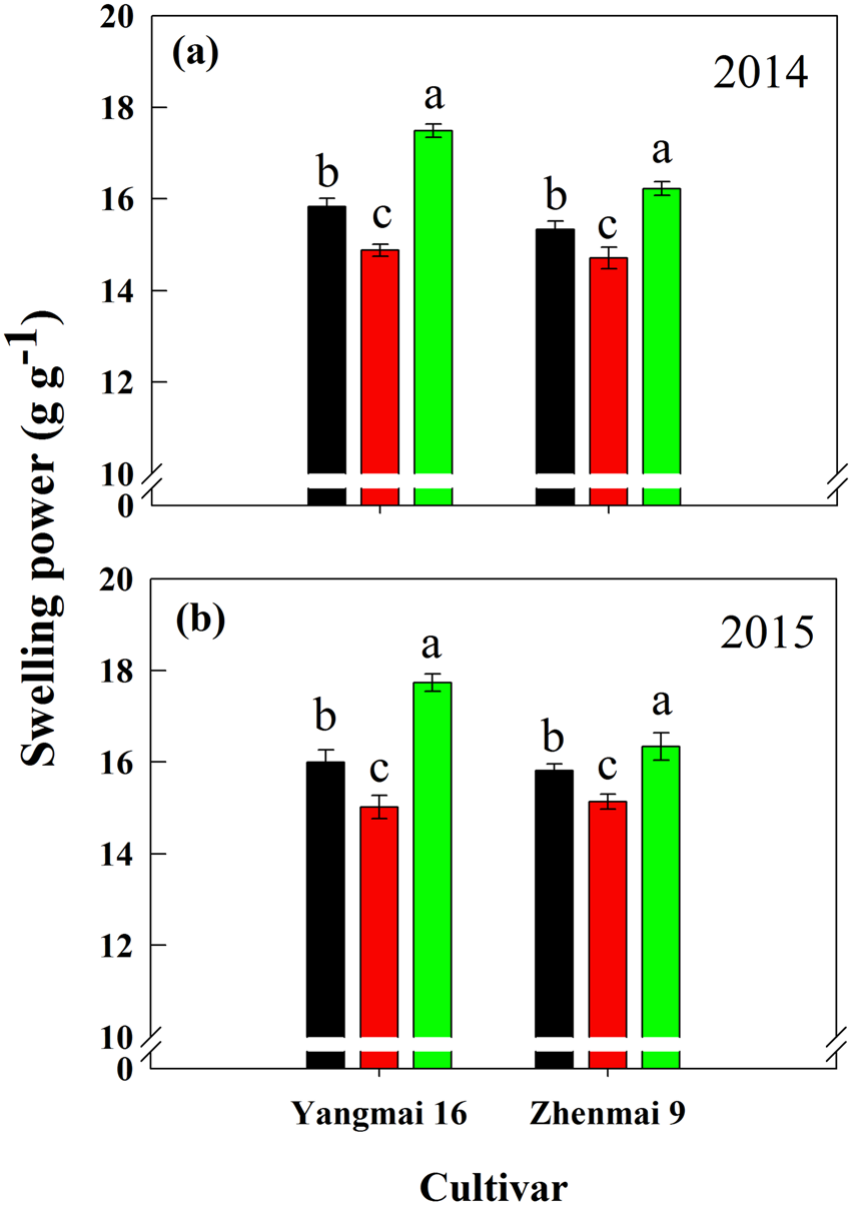
Swelling power for starches of wheat cultivars Yangmai 16 and Zhenmai 9 under various soil moisture treatments in 2014 (**a**) and 2015 (**b**). WW, MD, and SD represent well-watered, moderate soil-drought, and severe soil-drought treatments, respectively.

Swelling power assesses the extent of interactions between starch chains within amorphous and crystalline domains of a starch granule^30^. Soil drought greatly affected swelling power in both wheat cultivars (Fig. 8). In both cultivars, the MD treatment significantly decreased, whereas the SD treatment significantly increased, swelling power (Fig. 8a,b).

### Starch thermal properties

Structural stability and the loss of molecular (double helical) order in starch can be reflected by its gelatinization temperatures (T_0_, T_p_, and T_c_) and ∆H_gel_, respectively. Compared with that of the WW treatment, the MD-treated starch had a higher gelatinization temperature, increased gelatinization range (∆T), and lower gelatinization enthalpy (∆H_gel_). The SD treatment had the opposite effects. Furthermore, starch retrogradation was observed when the gelatinized samples were stored at 4 °C for 7 days (Table 4). The retrogradation enthalpy (∆H_ret_) and retrogradation percentage (%R) were significantly increased under the MD, while decreased under the SD treatment, for both cultivars and in both years (Table 4).

**Table 4:** Thermal properties of wheat starches under various soil moisture treatments^a^.

Year/Cultivar	Treatment	T_0_ (°C)^b^	T_p_ (°C)^b^	T_c_ (°C)^b^	∆T (°C)^b^	∆H_gel_ (J g^−1^)^b^	∆H_ret_ (J g^−1^)	%R
2014								
Yangmai 16	WW	59.9 ± 0.2b	63.0 ± 0.2b	67.7 ± 0.2b	7.9 ± 0.2a	10.5 ± 0.4a	1.89 ± 0.07b	18.03 ± 0.40b
	MD	60.6 ± 0.2a	63.7 ± 0.2a	68.9 ± 0.2a	8.3 ± 0.1a	9.1 ± 0.2b	2.11 ± 0.05a	23.20 ± 1.12a
	SD	59.2 ± 0.3c	62.6 ± 0.3b	66.9 ± 0.3b	7.8 ± 0.5a	11.0 ± 0.2a	1.67 ± 0.06c	15.12 ± 0.61c
Zhenmai 9	WW	60.4 ± 0.4a	64.2 ± 0.2a	69.3 ± 0.1b	8.9 ± 0.5a	9.5 ± 0.3b	2.08 ± 0.08a	21.99 ± 0.32b
	MD	60.8 ± 0.1a	64.4 ± 0.2a	69.7 ± 0.2a	8.9 ± 0.2a	8.3 ± 0.4c	2.23 ± 0.07a	26.83 ± 1.20a
	SD	60.2 ± 0.2a	63.6 ± 0.2b	68.8 ± 0.2c	8.6 ± 0.4a	10.4 ± 0.2a	1.77 ± 0.06b	16.95 ± 0.70c
2015								
Yangmai 16	WW	60.1 ± 0.4b	63.2 ± 0.2b	67.9 ± 0.1b	7.9 ± 0.3ab	10.7 ± 0.3b	1.83 ± 0.03b	17.22 ± 0.67b
	MD	60.8 ± 0.2a	63.9 ± 0.2a	69.2 ± 0.3a	8.4 ± 0.4a	9.3 ± 0.2c	2.01 ± 0.05a	21.57 ± 0.86a
	SD	59.2 ± 0.4c	62.4 ± 0.3c	66.6 ± 0.4c	7.4 ± 0.2b	11.4 ± 0.4a	1.61 ± 0.10c	14.14 ± 0.62c
Zhenmai 9	WW	60.2 ± 0.2a	64.7 ± 0.2a	69.2 ± 0.3b	9.0 ± 0.4a	10.2 ± 0.3b	2.01 ± 0.04b	19.67 ± 0.37b
	MD	60.6 ± 0.6a	64.4 ± 0.3a	69.8 ± 0.2a	9.2 ± 0.5a	8.8 ± 0.4c	2.11 ± 0.02a	23.94 ± 0.86a
	SD	59.5 ± 0.5b	63.5 ± 0.3b	68.1 ± 0.1c	8.6 ± 0.5a	11.3 ± 0.2a	1.82 ± 0.03a	16.10 ± 0.33c
Analysis of variance								
Year (Y)		1.18^ns^	1.58^ns^	1.49^ns^	0.05^ns^	23.26^**^	9.37^**^	44.17^**^
Cultivar (C)		10.61^**^	154.56^**^	312.93^**^	61.42^**^	34.01^**^	58.55^**^	128.59^**^
Treatment (T)		40.48^**^	63.24^**^	197.95^**^	8.13^**^	162.40^**^	139.47^**^	401.10^**^
Y × C		6.42^*^	0.18^ns^	3.93^ns^	1.14^ns^	5.03^*^	0.32^ns^	3.46^ns^
Y × T		1.37^ns^	2.87^ns^	9.74^**^	0.74^ns^	0.70^ns^	2.75^ns^	2.53^ns^
C × T		2.90^ns^	9.39^**^	14.94^**^	0.87^ns^	1.61^ns^	0.90^ns^	2.94^ns^

^a^Data are means ± standard deviation from three independent measurements, with different letters indicating significant statistical differences at the p ≤ 0.05 level in the same column and the same cultivar in the same year; ^b^To, onset temperature; Tp, peak temperature; Tc, conclusion temperature; ∆T, gelatinization range (Tc − To); ∆Hgel, gelatinization enthalpy; ∆Hret, retrogradation enthalpy; %R, retrogradation percentage. *^,^**F values significant at the *P* = 0.05 and *P* = 0.01 levels, respectively. ns denotes insignificant at the *P* = 0.05 level.

### Starch hydrolysis properties

**Figure 9:**
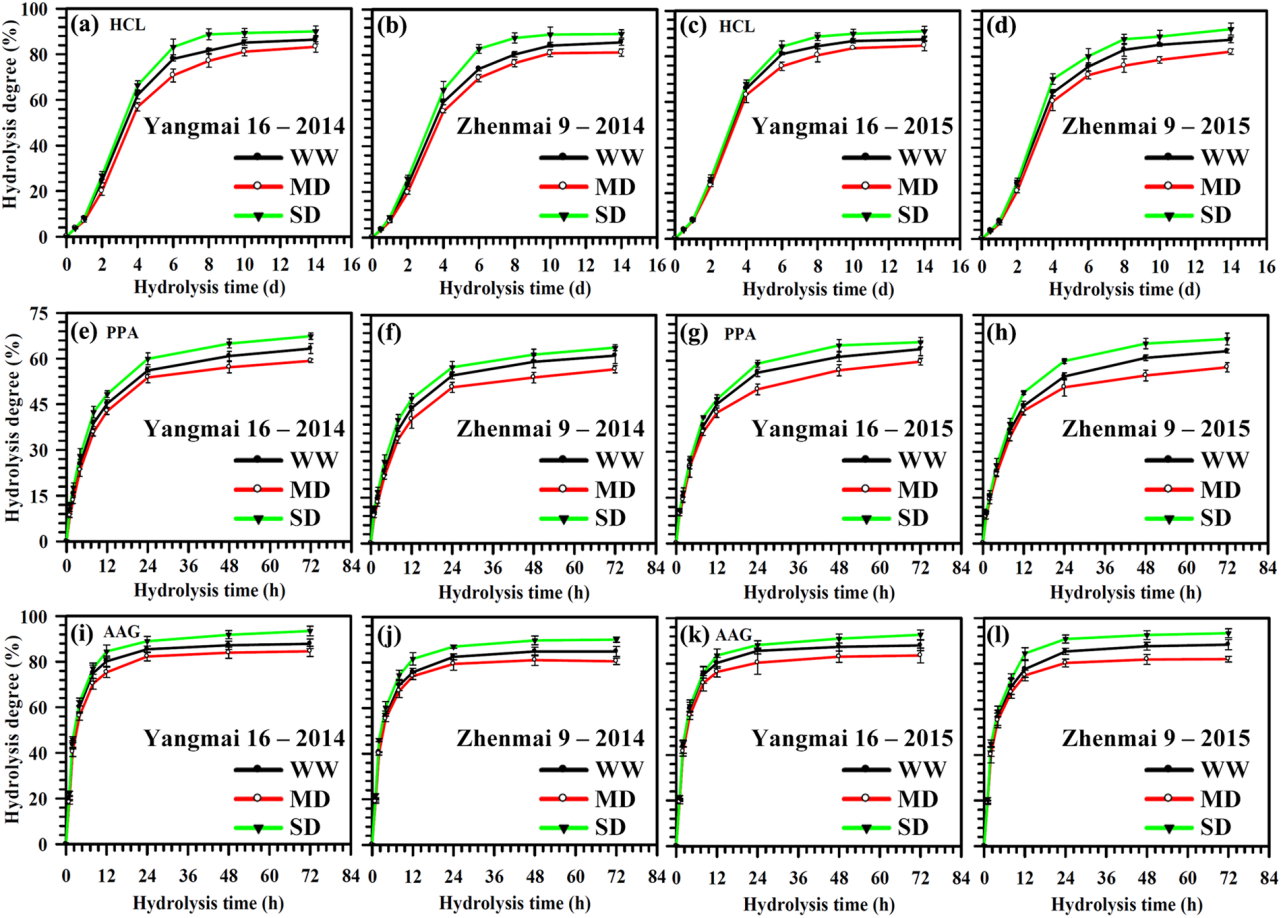
Degree of hydrolysis in starches, using HCl (**a**–**d**), PPA (**e**–**h**) or AAG (**i–l**), of wheat cultivars Yangmai 16 and Zhenmai 9 under various soil moisture treatments. WW, MD, and SD represent well-watered, moderate soil-drought, and severe soil-drought treatments, respectively. Vertical bars represent ± standard deviations of the mean (n = 3), where they exceed the size of the symbol.

In comparison with the WW treatment, the MD treatment significantly decreased, whereas the SD treatment significantly increased, the degree of starch hydrolysis under the three hydrolysis conditions (Fig. 9). Using HCl, the degree of hydrolysis was increased gradually with time (Fig. 9a–d). MD-treated starch showed greater resistance to HCl than WW starch, while the SD treatment showed less resistance (Fig. 9a–e). When hydrolysed by porcine pancreatic α-amylase (PPA) or *Aspergillus niger* amyloglucosidase (AAG), the two wheat cultivars exhibited relatively rapid initial rates from 0 to 8 h, and progressively decreased after 8 h (Fig. 9e–l). The results indicate that the higher amylose starch obtained from the MD treatment is more, whereas SD-treated starch is less, resistant to HCl, PPA and AAG than WW starch (Fig. 9a–i).

### Starch *in vitro* digestion properties

Native starches contained large amounts of resistant starch (RS) and lower levels of rapidly digestible starch (RDS) than gelatinized and retrograded starches, whereas retrograded starches had higher levels of slowly digestible starch (SDS) and lower levels of RDS than gelatinized starches, regardless of soil moisture levels (Table 5). Starches synthesized under MD conditions had lower RDS and higher RS contents than starches synthesized under WW conditions, especially in the gelatinized and retrograded forms (Table 5).

**Table 5:** *In vitro* digestion properties of wheat starches under various soil moisture treatments^a^.

Year/Cultivar	Treatment	Native starch			Gelatinized starch			Retrograded starch		
		RDS(%)^b^	SDS(%)^b^	RS(%)^b^	RDS(%)	SDS(%)	RS(%)	RDS(%)	SDS(%)	RS(%)
2014										
Yangmai 16	WW	19.6 ± 0.5a	12.7 ± 0.5b	67.7 ± 0.7b	60.4 ± 0.8b	0.8 ± 0.1b	38.8 ± 0.8b	49.2 ± 1.5b	4.3 ± 0.2b	46.5 ± 1.7b
	MD	19.2 ± 0.7a	9.8 ± 0.6c	71.0 ± 1.0a	57.7 ± 0.6c	1.4 ± 0.1a	40.9 ± 0.5a	46.1 ± 0.9c	5.2 ± 0.4a	48.7 ± 0.5a
	SD	20.5 ± 0.7a	13.9 ± 0.3a	65.6 ± 0.8c	62.1 ± 0.5a	0.8 ± 0.0b	37.1 ± 0.5c	51.8 ± 0.6a	3.6 ± 0.2c	44.6 ± 0.4c
Zhenmai 9	WW	17.9 ± 0.4b	12.3 ± 0.1b	69.8 ± 0.5b	59.6 ± 1.2a	0.8 ± 0.0b	39.6 ± 1.2b	49.4 ± 1.3a	4.1 ± 0.2b	46.6 ± 1.4ab
	MD	17.1 ± 0.5b	9.6 ± 0.5c	73.4 ± 1.0a	57.0 ± 0.7c	1.3 ± 0.1a	41.8 ± 0.8a	46.3 ± 0.9b	4.9 ± 0.4a	48.9 ± 0.6a
	SD	21.3 ± 1.0a	14.5 ± 0.5a	64.2 ± 0.5c	61.1 ± 0.8a	0.7 ± 0.0c	38.2 ± 0.8c	51.4 ± 1.0a	3.6 ± 0.2b	45.0 ± 1.2b
2015										
Yangmai 16	WW	19.3 ± 0.9ab	13.3 ± 0.2a	67.5 ± 1.0b	60.3 ± 0.4b	0.9 ± 0.0b	38.9 ± 0.5b	50.0 ± 0.6b	4.8 ± 0.1b	45.3 ± 0.5b
	MD	18.2 ± 0.2b	9.4 ± 0.8b	72.4 ± 0.9a	57.4 ± 0.1c	1.5 ± 0.1a	41.1 ± 0.1a	46.0 ± 0.5c	6.1 ± 0.6a	48.0 ± 0.5a
	SD	20.8 ± 0.5a	14.2 ± 0.2a	65.0 ± 0.3c	63.0 ± 0.5a	0.7 ± 0.0c	36.3 ± 0.5c	51.1 ± 0.1a	4.0 ± 0.4b	44.9 ± 0.5b
Zhenmai 9	WW	20.1 ± 0.3b	13.1 ± 1.1b	66.8 ± 0.9b	60.8 ± 0.5b	0.7 ± 0.0b	38.5 ± 0.5b	51.7 ± 0.7b	3.9 ± 0.4b	44.4 ± 1.0b
	MD	19.1 ± 0.4c	10.7 ± 0.5c	70.3 ± 0.8a	58.8 ± 0.5c	1.2 ± 0.0a	40.1 ± 0.5a	48.4 ± 0.6c	4.8 ± 0.5a	46.8 ± 1.1a
	SD	22.3 ± 0.2a	15.6 ± 0.4a	62.1 ± 0.6c	63.2 ± 0.3a	0.6 ± 0.0b	36.2 ± 0.3c	52.9 ± 0.1a	3.1 ± 0.1c	44.0 ± 0.2b
Analysis of variance										
Year (Y)		13.42^**^	4.31^*^	9.67^**^	65.81^**^	7.40^**^	61.45^**^	11.77^**^	0.21^ns^	10.99^**^
Cultivar (C)		0.01^ns^	9.64^**^	8.52^**^	11.94^**^	114.77^**^	17.70^**^	17.16^**^	18.50^**^	4.10^*^
Treatment (T)		74.88^**^	231.02^**^	245.28^**^	142.94^**^	790.16^**^	96.00^**^	141.09^**^	71.58^**^	56.29^**^
Y × C		27.80^**^	2.34^ns^	15.07^**^	52.11^**^	26.11^**^	45.95^**^	12.63^**^	20.48^**^	1.95^ns^
Y × T		0.35^ns^	0.50^ns^	0.82^ns^	30.27^**^	2.25^ns^	29.92^**^	0.99^ns^	2.91^ns^	1.20^ns^
C × T		8.56^**^	1.55^ns^	5.95^**^	24.06^**^	17.37^**^	22.61^**^	0.67^ns^	2.20^ns^	0.82^ns^

^a^Data are means ± standard deviation of three independent measurements, with different letters indicating significant statistical differences at the p ≤ 0.05 level in the same column and the same cultivar in the same year; b RDS, SDS, and RS represent rapidly digestible starch, slowly digestible starch, and resistant starch, respectively. *^,^**F values significant at the *P* = 0.05 and *P* = 0.01 levels, respectively. ns denotes insignificant at the *P* = 0.05 level.

### Relationship between functional properties and fine structure of starch

Pearson’s bivariate correlation analysis showed that amylose content, amylopectin long branch chain content (DP ≥ 37), average amylopectin chain length, and proportion of large granules had negative correlations with relative crystallinity, swelling power, ∆H_gel_, degree of hydrolysis (HCl, PPA, and AAG), and RDS content (*r* = −0.227 to −0.992, *P* < 0.05 or 0.01), but positively correlated with ∆H_ret,_ %R, and RS content (*r* = 0.245 to −0.977, *P* < 0.05 or 0.01) (see Supplementary Tables S1 and S2). Amylopectin short branch chain content (DP ≤ 24), degree of amylopectin branching, and proportion of small granules were positively correlated with relative crystallinity, swelling power, ∆H_gel_, degree of hydrolysis (HCl, PPA, and AAG), and RDS content (*r* = 0.227 to 0.984, *P* < 0.05 or 0.01), and were negatively correlated with ∆H_ret,_ %R, and RS content (*r* = −0.245 to −0.963, *P* < 0.05 or 0.01) (see Supplementary Tables S1 and S2).

## Discussion

It is well known that soil drought is the most important abiotic stress that causes significant yield loss worldwide^19,31–33^. We also observed that severe soil-drought decreased wheat grain yield (Fig. 1). However, our present results showed that moderate soil-drought, that is, plant water status was not severely inhibited (midday leaf water potential above −1.5 MPa) and plant could rehydrate overnight, could not only accelerate grain filling rate, but also increase grain weight and yield (Figs 1–2). The observation would have great significance in achieving the dual goal of increasing crop yield and saving water.

As starch in wheat endosperm contributes about 65–75% of the final dry weight of a grain^1^, the grain filling is actually a process of starch accumulation, the enzymes involved in starch synthesis play key roles in grain development and final product quality. Soil drought has been demonstrated to influence starch synthesis by regulating the activities of enzymes in starch biosynthesis, such as granule-bound starch synthase (GBSS), soluble starch synthase (SSS) and ADP-glucose pyrophosphorylase (ADGP-ppase)^21^. In this study, we observed that, compared with the WW treatment, both GBSS and SSS activities in wheat grains were significantly enhanced by the MD treatment, but significantly decreased by the SD treatment during the grain filling period for both cultivars in both years (Fig. 3). Consistent with the result, total starch accumulation in the grains was promoted by the MD treatment and decreased by the SD treatment relative to WW treatment (Table 1).

Starch granule size distribution largely affects the final product quality^34^. Wheat starch granules can normally be divided into two types: large granules (diameter > 10.0 μm) and small granules (diameter < 10.0 μm)^35^. Large and small starch granules exhibit different physicochemical properties due to their different chemical compositions (amylose content, amylose-lipid complex, and phosphorus content)^36^. Previous studies have shown that soil drought reduced the granule size in cereal crops^37,38^. However, our results showed that the response of wheat starch granule size to soil drought varied with the degree of water stress (Table 1, Fig. 4). In cereals, large granules have been associated with a higher proportion of amylose content than small granules, and related to GBSS^10,39–42^. The results suggest that the higher proportion of large granules and total starch accumulation in MD-treated grains is mainly due to the enhancement of GBSS activity, while SD treatment had the opposite effect (Fig. 3), which also contributes to a greater grain filling rate under the MD and a smaller grain filling rate under the SD treatment (Fig. 2).

Our results showed that, although both GBSS and SSS activities were significantly increased by the MD treatment, there was more increase in the proportion of amylose in MD-treated starch granules. A probable explanation is that the greater enhancement in GBSS activity than SSS activity contributes to fewer short chains in amylopectin, higher amylose content, and more amylopectin long chains in the MD-treated starch (Figs 3, 5 and 6; Tables 1–3). The results imply that the amylose synthesis which is mainly catalyzed by GBSS is more influenced by the soil drought degree than amylopectin synthesis which is mainly catalyzed by SSS^10,39–42^.

X-ray diffraction is a very effective method for measuring the crystal structure and regular molecular arrangement of native and processed starch samples^43^. The fine structure and properties of starch, such as molecular weight distribution, branch length of amylopectin short chains, and amylose-to-amylopectin ratio, profoundly influence starch relative crystallinity^44^. We found that the SD-treated starch, which showed the lowest amylose content, had the highest crystallinity, while the MD-treated starch, which showed the highest amylose content, had the lowest crystallinity (Fig. 7; Tables 2 and 3), in agreement with previous reports that crystallinity showed a significant negative correlation with amylose content^45^. Amylopectin is generally considered responsible for starch crystallinity, with amylose disrupting amylopectin crystalline packing^30^. Our results showed that relative crystallinity was correlated with starch fine structure (see Supplementary Table S1). Therefore, we speculate that the MD could increase, whereas the SD could decrease, amylose content, intermediate and long branch amylopectin chains, and the proportion of large granules in both cultivars, which might cause lower crystallinity under MD treatment and higher crystallinity under SD treatment.

Swelling power is an important parameter to assess the interaction between starch chains^30^. The differences in swelling power among the different soil moisture treatments in the present study could be attributed to the variation in amylose contents and levels of lipid-complexed amylose chains (Figs 5 and 8; Table 2). Amylose holds back swelling and maintains the integrity of swollen granules, and lipid-complexed amylose chains restrict both granular swelling and amylose leaching^46–48^. Moreover, we observed that swelling power was negatively correlated with amylopectin long branch chain content (DP ≥ 37), average amylopectin chain length, and proportion of large granules (see Supplementary Table S1). The results suggest that MD-treated starches had higher, while SD-treated starches had lower, amylose content, amylopectin long branch chain content, average amylopectin chain length, and proportion of large granules than WW starches (Tables 1–3), which might cause the lower swelling power under MD treatment and higher swelling power under SD treatment (Fig. 8).

Chemical composition is the major factor determining starch thermal properties. Amylose has been reported to have a high gelatinization temperature because its double helices require a high temperature and energy input to become disordered^28^. The degree of heterogeneity in crystallites within the starch granules has been shown to greatly affect ∆T^49^. ∆H_gel_ has been reported to decrease with the increase in amylose content, primarily reflecting the loss of double-helical order^50^. Amylose forms double-helical associations of 40–70 glucose units, while amylopectin crystallization occurs by association of the outermost short branches during retrogradation^51^. Higher levels of recrystallized domain could be attributed to a higher proportion of long B1 chains and higher average chain length^52^. MD-treated starches had a lower ∆H_gel,_ but higher ∆H_ret,_ ∆T and %R than WW-treated starches, mainly due to the higher amylose content and lower relative crystallinity of these samples, as determined by the GPC profile and XRD spectra, respectively (Figs 5–7; Tables 2–4), which was proved by the analysis of correlation between fine structure and thermal properties (see Supplementary Table S1).

Starch is usually hydrolysed by acid, alkali, or enzymes in biological and industrial processes, such as plant starch metabolism, mammalian digestion, fermentation, malting, or bioethanol production^53^. Many factors, such as amylose content, amylose-to-amylopectin ratio, crystalline structure, granule size, granule surface area, integrity, porosity, and the structural heterogeneity of granules might affect the susceptibility of starch to HCl, PPA, and AAG^54^. Previous studies showed that the degree of starch hydrolysis by amylase or acid is inversely related to the amylose content and proportion of large granules, which are properties associated with higher amylose content^39,40,55^. We also observed that the degree of hydrolysis (HCl, PPA, and AAG) was negatively correlated with amylose content and proportion of large granules (see Supplementary Table S1). However, we found that amylopectin fine structure also affected the degree of hydrolysis (HCl, PPA, and AAG), i.e., amylopectin long branch chain content (DP ≥ 37) and average amylopectin chain length had very significant and negative correlations with degree of hydrolysis (HCl, PPA, and AAG) (Figs 6 and 9; Table 3 and see Supplementary Table S1). The lower degree of hydrolysis in MD-treated starch than WW-treated starch accounted not only for its higher amylose content and higher proportion of large granules, but also for its higher amylopectin long branch chain content (DP ≥ 37) and average amylopectin chain length. The SD treatment resulted in the opposite traits, leading to a higher degree of hydrolysis.

*In vitro* digestion of starch using both porcine pancreatic α-amylase (PPA) and *Aspergillus niger* amyloglucosidase (AAG) is usually used to simulate the effects of hydrolysis in the small intestine and subsequent glycaemic responses^56^. RDS causes a rapid increase in blood glucose level after ingestion, whereas SDS releases glucose slowly and consistently over an extended time. RS which resists enzymatic hydrolysis is fermented in the large intestine releasing short chain fatty acids which are considered health benefits^57^. Inter- and intra-molecular hydrogen bonds in the starch chains can be disrupted when starch granules in water are exposed to heat, allowing the granules to swell and disintegrate. Therefore, the availability of starch chains to digestive enzymes increases during gelatinization. During the retrogradation of gelatinized starch, amylopectin recrystallizes to form crystallites, while amylose chains associate to form an amorphous matrix, which increases resistance to digestive enzymes. We found that the SD-treated starch, which showed the lowest amylose content, had the lowest RS content, while the MD-treated starch, which showed the highest amylose content, had the highest RS content (Figs. 3 and 5; Tables 2 and 5). This partly explains higher amylose content of the MD-treated starch showed lower degree of hydrolysis and SD treatment resulted in a higher degree of hydrolysis. Differences in the *in vitro* digestibility between different starches have been attributed to many coinciding factors, such as source, granule size, amylose content, amylopectin branch chain length distribution, degree of crystallinity, polymorphic composition, and granular pores, fissures, and channels^58^. Our results showed a significant correlation between wheat starch structure and digestion (see Supplementary Table S2). Interestingly, the correlation between the fine structure and SDS content in native starches was opposite to correlation between the fine structure and SDS content in gelatinized and retrograded starches (see Supplementary Table S2). In this study, starch composition and physicochemical properties, such as total starch and amylose contents, amylopectin chain length distribution, relative crystallinity, granule size, and other thermal properties, were altered by the different soil moisture treatments, might together result in the native starches showing different digestion properties.

## Conclusion

Compared with the WW treatment, the MD treatment could increase total starch accumulation in the grain, the proportion of large starch granules, the amylose and amylopectin long branch chain contents (DP ≥ 37), and the average amylopectin branch chain length, but decrease the amylopectin short branch chain content, and the degree of amylopectin branching. The MD treatment exhibited a lower gelatinization enthalpy, pasting viscosity, and swelling power, but a higher gelatinization temperature, retrogradation enthalpy, retrogradation percentage, pasting peak time, and pasting temperature. The MD treatment could also increase starch resistance to acid and amylase hydrolysis. Gelatinized and retrograded starches synthesized under MD conditions had lower RDS content and higher RS content. The SD treatment showed opposite effects. Substantial enhancement in GBSS activity under the MD enhanced amylose synthesis, whereas substantial reduction in GBSS activity under the SD decreased it. Amylose synthesis was more sensitive to soil drought than amylopectin synthesis in wheat grains. Moderate soil-drought, i.e., midday leaf water potential was above −1.5 MPa and plant could rehydrate overnight, could improve starch molecular structure and functional properties in the grain.

## Methods

### Plant materials and treatments

The experiment was conducted at a research farm of Yangzhou University, Jiangsu Province, China (32°30′ N, 119°25′ E, 21 m altitude) during two wheat growing seasons, November 2013–June 2014 and November 2014–June 2015. Two cultivars currently used in local production, Yangmai 16 and Zhenmai 9, with the protein content of 14.2% and 12.5%, respectively (measured by Infratec Food and Feed analyser, FOSS TECATOR, Sweden), were grown in porcelain pots, with twenty seeds per pot. Each porcelain pot (30 cm in height and 25 cm in diameter, 14.72 L in volume) was filled with 18 kg sandy loam soil [Typic fluvaquents, Entisols (U.S. taxonomy)] that contained 20.2 g kg^−1^ organic matter, 105 mg kg^−1^ alkali hydrolysable N, 34.2 mg kg^−1^ Olsen-phosphorus and 68.0 mg kg^−1^ exchangeable potassium. On the day of sowing (3 November), 1 g N as urea and 0.2 g P as single superphosphate were mixed into the soil in each pot. N as urea was also top-dressed into each pot at the rate of 0.4 g and 0.6 g at 30 days after sowing (DAS) and 112 DAS, respectively. At the 3 leaves unfolded stage (2-digit code: 13)^59^, the plants were thinned to eight plants per pot (equivalent to a density of 163 plants m^−2^). The plants were watered daily by hand to maintain a soil water content close to field capacity (soil moisture content 0.189 g g^−1^) until the stage at 7 tillers in the main stem (2-digit code: 27)^59^ when soil drought treatments were initiated. The flowering dates of both cultivars were similar, with initial flowering dates of April 9–10 in 2014, and April 11–13 in 2015. The air temperature during the grain filling period (April–May) in both study years was measured at a weather station close to the experimental site, and was shown in Supplementary Fig. S1.

The experiment was a two-by-three factorial design (two cultivars, three levels of soil moisture). Each treatment comprised 48 pots as replicates in a completely randomized block design. From 7 tillers in the main stem to maturity (1-digit code: 9)^59^, three levels of soil water potential (ψ_soil_) were imposed on plants by controlling water application. The Well-watered (WW) treatment was maintained at −20 ± 5 kPa (soil moisture content, 0.155 g g^−1^), while the moderate soil-drought (MD) treatment was maintained at −40 ± 5 kPa (soil moisture content. 0.119 g g^−1^) and the severe soil-drought (SD) treatment was kept at −60 ± 5 kPa (soil moisture content. 0.091 g g^−1^). The soil water potential was monitored at a soil depth of 15–20 cm. A tension meter consisting of a 5-cm-long sensor (Soil Science Research Institute, China Academy of Sciences, Nanjing, China) was installed in each pot to monitor soil moisture. Tension meter readings were recorded every 4 h between 06:00 and 18:00, daily. When readings dropped to designated values, 150, 120, or 90 mL of tap water were added to WW, MD, and SD plants, respectively. The soil water potential maximally rose to −15, −35, or −55 kPa for WW, MD, and SD, respectively, after re-watering. Total water application during the whole growing season and the dynamic changes of soil water potential were shown in Supplementary Fig. S2. The pots were placed in a field and sheltered during the rain using a removable polyethylene shelter.

### Sampling and determination of grain filling rate

A total of 200 spikes that headed on the same day were chosen and tagged for each treatment. Fifteen tagged spikes from each treatment were sampled at 6-day intervals from anthesis (1-digit code: 6) to maturity (1-digit code: 9)^59^ (42 DAA) for both cultivars since they had very similar growth periods. The sampled spikes were divided into three groups (5 spikes each) as subsamples for the measurement of SSS and GBSS activities, and grain weight. All grains from each spikelet were removed. The sampled grains were frozen in liquid nitrogen for 2 min and then stored at −80 °C for enzymatic measurements. The grain filling process was fitted by Richards’s Growth Equation (Richards 1959) according to the method described by Zhu *et al*.^60^.

1 $$W=\frac{A}{{(1+B{e}^{-kt})}^{\frac{1}{N}}}$$

The grain filling rate (*G*) was calculated as the derivative of equation (1)

2 $$G=\frac{AkB{e}^{-kt}}{N{(1+B{e}^{-kt})}^{\frac{(N+1)}{N}}}$$

where *W* is grain weight, *A* denotes the final grain weight; *t* presents the time after anthesis (days); and *B*, *k*, and *N* are the regression coefficients. The period of active grain filling is defined as the time interval taken for *W* to change from 5% (*t*_1_) to 95% (*t*_2_) of *A*. The average rate of grain filling during this period was calculated from *t*_1_ to *t*_2_.

Plants in 10 pots of each treatment were harvested at maturity (1-digit code: 9) for the determination of grain yield. Yield components, i.e., number of spikes, grains per spike, and 1000-grain weight were determined from 50 plants in each treatment.

### Measurement of leaf water potentials

Measurements of leaf water potentials of the upmost fully-expanded leaves on stems were made at predawn (06:00 h) and midday (11:30 h) at 0, 12, 24, 36, 48, 60, 72 days after soil moisture treatment, respectively. Three pressure chambers (Model 3000, Soil Moisture Equipment Corp., Santa Barbara, CA, USA) were used for leaf water potential measurement, with six leaves for each treatment.

### Enzyme extraction and assays

Three replications were performed for each treatment and for all measurements below. The method for soluble starch synthase (SSS) and granule bound starch synthase (GBSS) extract was described by Zhu *et al*.^61^ the sampled grains (180-220 mg) were homogenized in a pre-cooled mortar containing 1 mL of extraction buffer comprising, 100 mM HEPES-NaOH (pH 7.6), 5 mM MgCl2, 5 mM dithiothreitol (DTT), 2 mM EDTA, 12.5% (v/v) glycerol, and 5% (w/v) insoluble polyvinylpyrrolidone 40. The homogenate was centrifuged at 12000 × *g* for 10 min, and then the supernatant was collected for the SSS activity assay. The supernatant was re-suspended in 1 mL of extraction buffer for GBSS activity analysis. The SSS and GBSS activities in the grains were determined by the method of Wang *et al*.^23^. All chemicals and enzymes used for enzymatic measurement were from Sigma Chemical Company (St Louis, MO, USA). All the enzyme activities were expressed as nmol mg^−1^ protein min^−1^.

### Total starch determination and isolation

Total starch accumulation in the wheat grains was determined using the method described by Zhu *et al*.^62^ after a minimum storage period, and was defined as the sum of amylose and amylopectin. Briefly, the sampled grains were ground in mortar, and then the powder was degreased twice with anhydrous ether. A 100 mg fraction of each sample was used to determine amylose and amylopectin contents. A calibration curve was derived using pure amylose and amylopectin from potato and maize, respectively.

Starch was isolated from the peeled grains according to the method of Gao *et al*.^53^ with some modifications. Briefly, wheat flour of 10 g from each treatment was steeped in NaOH solution (pH = 9.5) with 50 mg**·**g^−1^ alkaline protease at 42 °C for 24 h to remove protein. The starch slurry was through eight layers of cotton gauze and 200-mesh sieves, and the filtrate was collected. The filtrate was centrifuged at 3,000 × *g* for 10 min, and then the supernatant was discarded. The faintly colored supernatant liquid was carefully scraped off, while the remaining white precipitate was re-suspended with 20 mL of deionized water, centrifuged at 3,000 × *g* for 10 min, and the supernatant was again removed. The aforementioned centrifugal steps were repeated five times to ensure thorough removal of impurities. Finally, the starch was dried at 30 °C at ambient pressure and the dried starch was put through a 200-mesh sieve, and then stored in a closed dry container until further analysis.

### Granule size distribution

The particle size characteristics of the starch were determined using an MS-2000 laser particle size analyser (Malvern, England). The instrument uses laser light scattering to measure sizes between 0.2 and 2000 μm. The dispersed phase was absolute ethyl alcohol. Starch granule size distributions were measured using the native instrument software and expressed as percentages.

### Apparent amylose content determination and molecular weight distribution analysis

The apparent amylose content in starch was determined using the iodometric method described by Man *et al*.^63^. Briefly, starch was defatted in methanol/water (85:15, v/v) at 65 °C for 1 h and dissolved in urea dimethyl sulfoxide (UDMSO) solution at 95 °C for 1 h. The starch-UDMSO solution was treated with I2-KI solution. The apparent amylase content was calculated from the absorbance at 620 nm by reference to a calibration curve which was derived using pure amylose and amylopectin from potato and maize, respectively.

Starch was deproteinised with protease and sodium bisulfite, and then debranched according to the methods of Li *et al*.^64^ and Tran *et al*.^65^. The molecular weight distribution of the debranched starch was determined by gel-permeation chromatography (GPC) using a PL-GPC 220 system (Polymer Laboratories Varian, Inc.; Amherst, MA), which included three columns (PL110-6100, -6300, and -6525) and a differential refractive index detector, according to the method of Cai *et al*.^66^. The dimethyl sulphoxide (DMSO) containing 0.5 mM NaNO_3_ was used as eluent system at a flow rate of 0.8 mL min^−1^. The column oven temperature was controlled at 80 °C. Standard dextrans (molecular weights: 2800, 18500, 111900, 410000, 1050000, 2900000 and 6300000) were used for column calibration and, on the basis of the standards, and the relative molecular weight (molecular size) was calculated.

### Amylopectin chain length distribution

The chain length distributions in the debranched samples were analysed using the method of Lin *et al*.^67^, using a high-performance anion-exchange chromatograph (HPAEC; Thermo ICS-5000, Thermo Corp., Sunnyvate, CA) equipped with a pulsed amperometric detector, guard column, CarboPacTM PA200 analytical column, and AS-DV autosampler. Briefly, the starch was deproteinized with protease and sodium bisulfite, and then debranched with isoamylase. Debranching was terminated by adding NaOH and heating to 80 °C. The sample was centrifuged at 10,000 × *g* for 10 min, filtered (0.22-μm nylon filter) and injected into an HPAEC system with a pulse amperometric detector (PAD) system. Eluent A was 150 μM NaOH, and eluent B was 150 μM NaOH with 500 μM sodium acetate. The gradient of eluent B was 35% from 0 to 2 min, increased from 35% to 60% for 15 min and from 60% to 80% for 13 min, maintained 80% for 10 min, and finally reduced from 80% to 30% for 0.2 min. The separations were carried out at 25 °C with a flow rate of 0.5 mL min^−1^. Maltohexaose was used as a standard. The chain length distribution was characterized as a percentage of the total peak area.

### X-ray powder diffraction (XRD) analysis

Starch XRD patterns were obtained using a D8 Advance diffractometer (Bruker-AXS, Germany). The diffractometer was operated at 200 mA and 40 kV. The scanning region of the diffraction angle (2θ) ranged from 3° to 40° at a step size of 0.02° and counting time of 0.8 s. XRD analysis and determination of the relative crystallinity (%) of starch were carried out following the method described by Wei *et al*.^68^. Before measurements, all specimens were stored in a desiccator for one week at a constant humidity (relative humidity = 75%) maintained by saturated brine.

### Differential scanning calorimetry (DSC) analysis

Starch (5.0 mg) was precisely weighed and mixed with distilled water (15 μL). The mixture was sealed in an aluminium pan overnight at 4 °C. After equilibrating for 1 h at room temperature, the starch sample was then heated from 25 to 130 °C at a rate of 10 °C min^−1^ using a differential scanning calorimeter (DSC 200 F3, Netzsch Instruments NA LLC; Burlington, MA).

### Determination of starch swelling power

Starch (40 mg) was placed into a centrifuge tube with double-distilled H_2_O (1 mL). The samples were heated to a constant temperature of 90 °C in a shaking water bath for 1 h and then centrifuged at 3,000 × *g* for 15 min. The supernatant was removed, and the starch deposit was weighed in the centrifuge tube. The residue was dried to a constant weight at 70 °C for 48 h, and reweighed. Swelling power (g g^−1^) was calculated using the following equation:

$${\rm{Swelling}}\,{\rm{power}}=({{\rm{m}}}_{2}-{{\rm{m}}}_{1})/({{\rm{m}}}_{3}-{{\rm{m}}}_{1})$$

where m_1_ is the weight of the centrifuge tube, m_2_ is the total weight of the centrifuge tube and undried residue, and m_3_ is the total weight of the centrifuge tube and dried residue.

### Determination of hydrolysis degree

Starch was hydrolysed using hydrochloric acid (HCl), porcine pancreatic α-amylase (PPA, A3176; Sigma-Aldrich), or *Aspergillus niger* amyloglucosidase (AAG, A7095; Sigma-Aldrich) following the method described by Huang *et al*.^69^. The hydrolysis time points were 1, 2, 4, 8, 12, 24, 48, and 72 h for PPA and AAG, and 0.5, 1, 2, 4, 6, 8, 10, and 14 days for HCl. After hydrolysis, the starch slurry was quickly centrifuged (8,000 × *g*) at 4 °C for 5 min. The supernatant was used to measure soluble carbohydrates using the anthrone-H_2_SO_4_ method to quantify the degree of hydrolysis.

### *In vitro* starch digestion

*In vitro* starch digestion was analysed using the method described by Huang *et al*.^69^. Native starch (10 mg) was mixed with distilled water (2 mL) in a centrifuge tube and heated at 98 °C for 12 min to prepare gelatinised starch. The gelatinised starch was stored at 4 °C for 36 h to prepare retrograded starch. The starch (10 mg) was then incubated in enzyme solution (2 mL, 20 μM sodium phosphate buffer pH 6.0, 6.7 μM NaCl, 0.01% NaN_3_, 2.5 μM CaCl_2_, 4 U PPA (Sigma A3176), 4 U AAG (Megazyme E-AMGDF)) and digestion was conducted in an Eppendorf Thermo Mixer at 37 °C with continuous shaking (2,000 × *g*) for 20 and 120 min. Enzyme treatment was terminated by adding 0.1 M HCl (240 mL) and 50% ethanol (2 mL) and centrifuging (14,000 × *g*, 5 min). The glucose content in the supernatant was determined using the D-Glucose (GOPOD Format) assay kit (Megazyme, K-GLUC). Starch nutritional fractions, based on the rate of hydrolysis, were denoted as rapidly digestible starch (RDS, digested within 20 min), slowly digestible starch (SDS, digested in 20–120 min), and resistant starch (RS, undigested after 120 min).

### Statistical analysis

Statistical analyses of the results for variance were carried out using the SAS/STAT statistical analysis package (version 9.2, SAS Institute; Cary, NC, USA). The statistical model used included sources of variation due to replication, year, variety, soil moisture treatment, and the interaction of year × variety, year × treatment and cultivar × treatment. Data from each sampling date were analysed separately, and means were tested using least significant differences at the *P*_0.05_ level (LSD_0.05_).

## Electronic supplementary material

Supplementary Information
