# Supplementary material for: Comparison of Structural and Functional Properties of Wheat Starch Under Different Soil Drought Conditions
Source: Sci Rep. 2017 Sep 26;7:12312. doi: 10.1038/s41598-017-10802-3 (PMC5615046; doi:10.1038/s41598-017-10802-3)
Supplement: Supplementary file 1 — Supplementary Information [file 41598_2017_10802_MOESM1_ESM.pdf]

Supporting Information

Comparison of Structural and Functional Properties of Wheat Starch Under Different Soil Drought Conditions

Weiyang Zhang [a], Junfei Gu [a], Zhiqin Wang [a], Cunxu Wei [a], Jianchang Yang [a]*, Jianhua Zhang[b]

[a] Jiangsu Key Laboratory of Crop Genetics and Physiology/Co-Innovation Center for Modern Production Technology of Grain Crops, Yangzhou University, Yangzhou, Jiangsu, China

[b] School of Life Sciences and State Key Laboratory of Agrobiotechnology, The Chinese University of Hong Kong, Hong Kong, China

* Corresponding Author

Jianchang Yang. Address: College of Agriculture, Yangzhou University, Yangzhou 225009, China. Telephone/Fax: +86 514 8797 9317. E-mail: jcyang@yzu.edu.cn; jsyzyjc@126.com

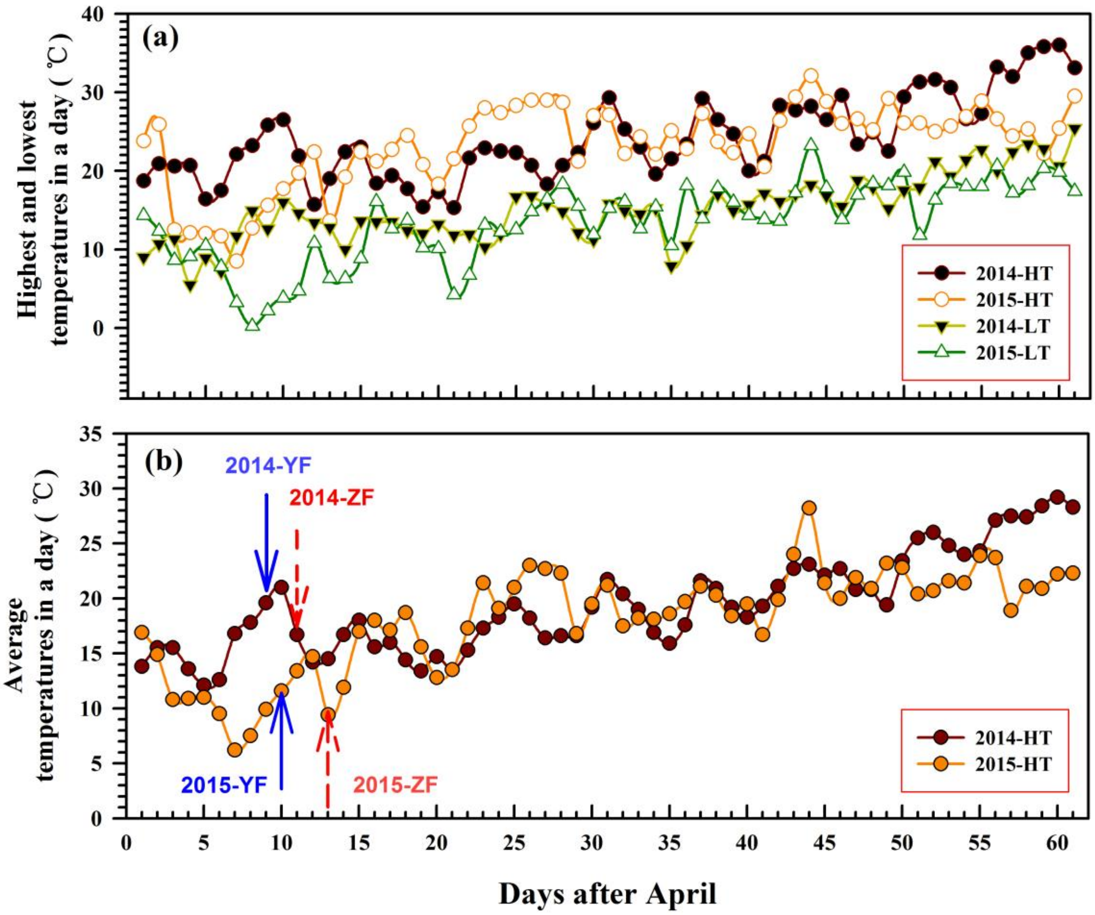

Supplementary Fig. S1. Temperatures during the grain-filling stage (April-May) in 2014 and 2015. HT, LT and AT indicate the highest temperatures, lowest temperatures and average temperatures in a day, respectively. Arrows indicate the flowering date of the two cultivars in two years. YF and ZF indicate the Yangmai 16 flowered and Zhenmai 9 flowered, respectively.

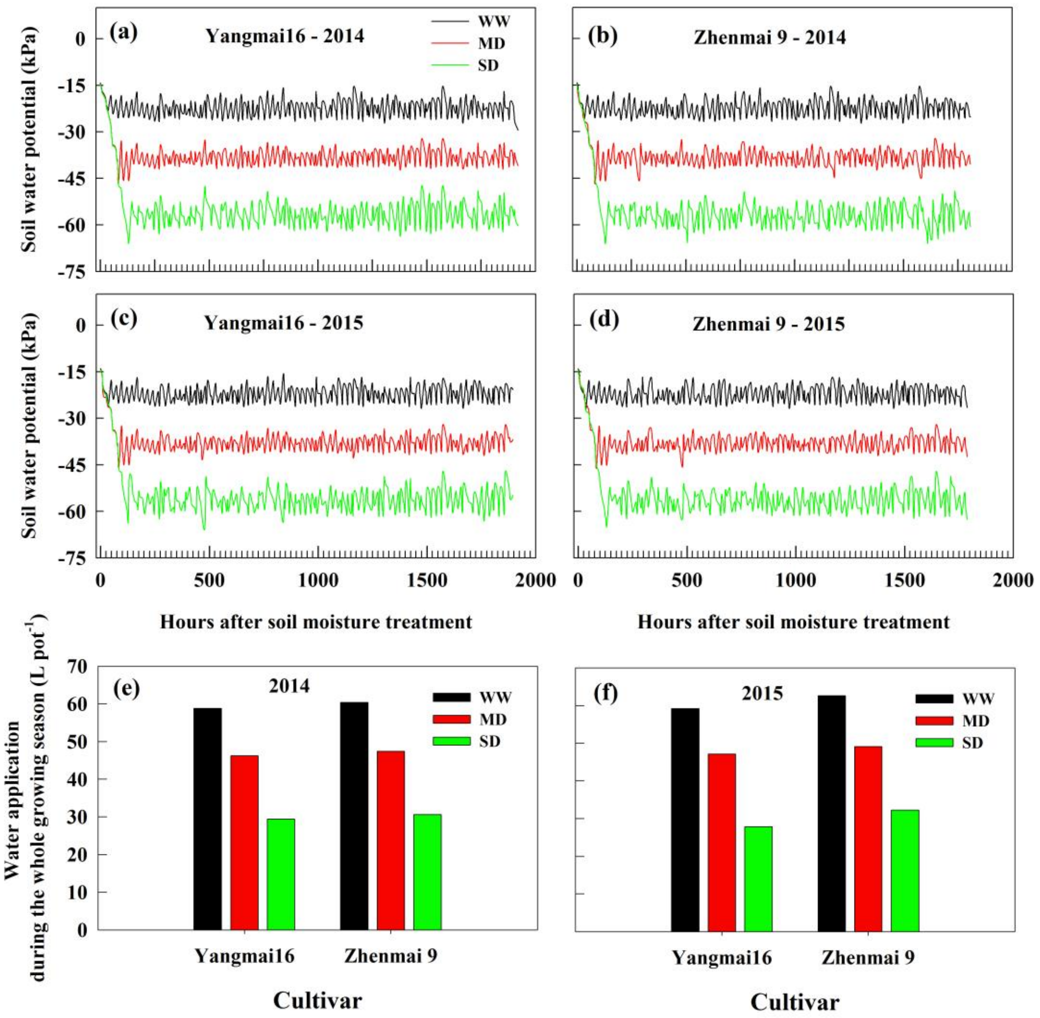

Supplementary Fig. S2. Soil water potential (a-d) after soil moisture treatments and water application during whole growing (e, f) of the wheat cultivars Yangmai 16 and Zhenmai 9 under various soil moisture treatments in 2014 and 2015. WW, MD, and SD represent well-watered, moderate soil-drought, and severe soil-drought treatments, respectively. Vertical bars represent ± standard deviations of the mean (n = 3), where they exceed the size of the symbol.

Supplementary Table S1. Correlation analysis between fine structure and crystallinity, swelling power, thermal properties, and hydrolysis properties of starches.[a]

Correlated with	crystallinity	Swelling power	Thermal properties[d]			Hydrolysis properties[e]		
			ΔH_{gel}	ΔH_{ret}	R (%)	HCL	PPA	AAG
Amylose content[b]	–0.968**	–0.898**	–0.971**	0.901**	0.943**	–0.973**	–0.985**	–0.992**
Branching degree of amylopectin[b]	0.916**	0.850**	0.918**	–0.920**	–0.939**	0.949**	0.932**	0.937**
Proportion of DP ≤ 24 in amylopectin[c]	0.939**	0.919**	0.962**	–0.925**	–0.956**	0.931**	0.979**	0.984**
Proportion of DP ≥ 37 in amylopectin[c]	–0.896**	–0.899**	–0.961**	0.923**	0.959**	–0.921**	–0.959**	–0.970**
Average chain length of amylopectin[c]	–0.969**	–0.898**	–0.961**	0.893**	0.932**	–0.976**	–0.979**	–0.990**
Proportion of large granules (d > 10 μm)	–0.576*	–0.227	–0.411	0.245	0.338	–0.555	–0.475	–0.458
Proportion of small granules (d < 10 μm)	0.576*	0.227	0.411	–0.245	–0.338	0.555	0.475	0.458

[a] * and** indicate the significance at $p < 0.05$ and $p < 0.01$ level (n=12), respectively.

[b] Molecular weight distribution of isoamylase-debranched starch is determined by gel-permeation chromatography.

[c] Chain length distribution of amylopectin is determined by high-performance anion-exchange chromatography.

[d] H_{gel}, gelatinization enthalpy; $\Delta H_{ret,}$ retrogradation enthalpy; %R, retrogradation percentage.

[e] Starch is hydrolyzed using hydrochloric acid (HCl), porcine pancreatic a-amylase (PPA) or Aspergillus niger amyloglucosidase (AAG).

Supplementary Table S2. Correlation analysis between molecular structure and *in vitro* digestion of starches.[a]

Correlated with	Native starch			Gelatinized starch			Retrograded starch		
	RDS (%)	SDS (%)	RS (%)	RDS (%)	SDS (%)	RS (%)	RDS (%)	SDS (%)	RS (%)
Apparent amylose content	–0.886**	–0.920**	0.942**	–0.946**	0.819**	0.952**	–0.872**	0.776**	0.868**
Amylopectin short branch-chain content[b]	0.904**	0.941**	–0.963**	0.954**	–0.836**	–0.958**	0.900**	–0.793**	–0.898**
Amylopectin long branch-chain content[b]	–0.780**	–0.805**	0.828**	–0.750**	0.685*	0.752**	–0.727**	0.584*	0.752**
Amylose content[b]	–0.902**	–0.941**	0.962**	–0.969**	0.846**	0.974**	–0.910**	0.815**	0.902**
Branching degree of amylopectin[b]	0.879**	0.910**	–0.933**	0.890**	–0.795**	–0.893**	0.850**	–0.735**	–0.856**
Proportion of DP 6-12 in amylopectin[c]	0.869**	0.897**	–0.920**	0.951**	–0.785**	–0.962**	0.882**	–0.704**	–0.951**
Proportion of DP 13-24 in amylopectin[c]	0.813**	0.836**	–0.860**	0.891**	–0.738**	-0.903**	0.807**	–0.713**	–0.807**
Proportion of DP 25-36 in amylopectin[c]	–0.761**	–0.877**	0.862**	–0.887**	0.814**	0.885**	–0.839**	0.825**	0.796**
Proportion of DP ≥ 37 in amylopectin[c]	–0.855**	–0.836**	0.878**	–0.905**	0.715**	0.923**	–0.816**	0.651*	0.850**
Average chain length of amylopectin[c]	–0.906**	–0.926**	0.955**	–0.968**	0.823**	0.977**	–0.904**	0.808**	0.898**
Relative crystallinity	0.848**	0.910**	–0.920**	0.925**	–0.814**	–0.929**	0.873**	–0.850**	–0.832**
Proportion of large granules (d > 10 μm)	–0.622*	–0.692*	0.689*	–0.552	0.696*	0.519	–0.260	0.773**	0.634*
Proportion of small granules (d < 10 μm)	0.622*	0.692*	–0.689*	0.552	–0.696*	–0.519	0.260	–0.773**	–0.634*

[a] * and** indicate the significance at $p < 0.05$ and $p < 0.01$ level (n=12), respectively.

[b] Molecular weight distribution of isoamylase-debranched starch is determined by gel-permeation chromatography.

[c] Chain length distribution of amylopectin is determined by high-performance anion-exchange chromatography.
